# Women's perspectives on human milk banking in Ghana: results from a cross-sectional study

**DOI:** 10.3389/fpubh.2023.1128375

**Published:** 2023-05-25

**Authors:** Cecilia Obeng, Frederica Jackson, Salome Amissah-Essel, Christiana Nsiah-Asamoah, Cydne A. Perry, Ines Gonzalez Casanova, Emmanuel Obeng-Gyasi

**Affiliations:** ^1^Department of Applied Health Science, School of Public Heath, Indiana University, Bloomington, IN, United States; ^2^Department of Health, Physical Education and Recreation, University of Cape Coast, Cape Coast, Ghana; ^3^Department of Clinical Nutrition and Dietetics, University of Cape Coast, Cape Coast, Ghana; ^4^Department of Built Environment, North Carolina Agricultural and Technical State University, Greensboro, NC, Canada; ^5^Environmental Health and Disease Laboratory, North Carolina Agricultural and Technical State University, Greensboro, NC, Canada

**Keywords:** human milk banking, Ghana, quantitative and qualitative analysis, population needs assessment, health

## Abstract

**Background:**

Although political and academic interest exists in Ghana to include human milk banks (HMB) into current maternal and child health programs, efforts to establish a HMB have not yet been subjected to any real empirical inspection with the view toward implementation. Furthermore, views toward the establishment of a HMB in Ghana have not been assessed among Ghanaian women. The aims of the current study were to examine Ghanaian women's views about HMB, and to investigate women's willingness to donate to a HMB.

**Methods:**

Quantitative and qualitative responses were received from Ghanaian females (*n* = 1,270) aged 18+ years. Excluding outliers and missing data (*n* = 321), a final sample of 949 was retained for final analysis. Chi-square tests and logistic regression analysis were computed on quantitative data; Thematic analysis was performed on the qualitative responses.

**Results:**

In our sample, 64.7% of respondents indicated that Ghana is ready for a HMB. The majority (77.2%) were willing to donate milk, and 69.4% believed that donating to the HMB would favor their child. The main concerns for the unwillingness to donate excess milk included: (i) the idea of HMBs as strange/bizarre (*n* = 47), (ii) fear of infections (*n* = 15), (iii) religious beliefs (*n* = 9), and (iv) insufficient information (*n* = 24). This study serves as the first step toward the development of a HMB in Ghana.

**Conclusions:**

Overall, Ghanaian women support the building of a HMB to enhance infant nutrition and reduce childhood morbidity and mortality.

## 1. Introduction

Despite progress made on reducing infant morbidity and mortality, infant deaths remain a major global health concern, especially in low-income settings. Recent data on infant mortality (2023) estimates global infant deaths at 26 per 1000 live births (1). In terms of neonatal deaths globally, an estimated 2.3 million newborn deaths occurred in 2021, which translates to 6, 400 neonates dying each day (2). There are great disparities in these deaths, where children born in Sub-Saharan Africa (SSA) are ten times more likely to die in the first 4 weeks after birth compared to those born in high-resource settings (3). In Ghana, infant mortality (2023) is estimated at 30.8 deaths per 1000 live births (4), with the national neonatal mortality rate (2021) being 23 per 1,000 live births (5). About 48% of under-five mortality in the country is attributed to neonatal mortality (6), and to illustrate, it is reported that a newborn dies every 15 min in the country (7–9) which accounts for up to 70 newborn deaths a day (10). Premature birth is a leading cause of newborn death (10, 11) with one in seven babies born before 37 weeks of gestation (12).

Evidence from the Ghana Demographic and Health Survey (2014) reports a 10% rate of low birthweight (LBW) in Ghanaian newborns (6, 13). However, the prevalence of LBW in parts of the country appears to be higher. For instance, 26% LBW was reported in a sample of 419 Ghanaian infant-mother pairs in Northern Ghana (14) and 21% LBW was reported in the Ashanti region of Ghana (15). To our knowledge, there is no available national data on the prevalence of preterm delivery (13) however, some existing studies shed light on the rates of premature births in the country. A study conducted in Ho (Volta Region) examining 680 health records of Ghanaian women estimated preterm delivery at 14.1% (13). Another retrospective descriptive study carried out in Korle-Bu Teaching Hospital in Accra (Ghana's Capital) examined 7,801 birth records of Ghanaian women who delivered between January 1 and December 31 2015 (16). The researchers report 18.9% occurrence of preterm delivery in their sample. Further, a study by Afeke et al. (17) examined 8,279 delivery records from the pediatrics unit of the Volta Regional Hospital spanning the time periods November 2011 to June 2016 (17). Low birthweight in the sample was 10.7%; the study showed that preterm babies had a 4% chance of survival within 7 days of birth compared to the 96% survival rate for term babies (17). Moreover, a prospective cohort study conducted at the Kinatampo Health Research center serving 4 district hospitals and 69 health facilities investigated neonatal mortality in a sample of 22, 906 records (18). The study revealed 16% low birthweight and 30.5 neonatal deaths per 1000 live births (18).

In response to the alarming rates of neonatal mortality globally and locally, researchers have uncovered the crucial benefits of human breastmilk for neonatal survival. Breastmilk has been shown to prevent infant deaths due to its capacity to mitigate newborn infections and malnutrition that are among the main causes of infant deaths in resource-poor settings. In this regard, evidence suggests that current feeding practices for infants in Ghanaian NICU's involves the mother expressing milk or directly breastfeeding the infant, if possible (12, 19). Because feeding NICU babies in Ghana relies predominantly on the availability of mother's breastmilk, this presents as a challenge for mothers who experience delayed lactation and low breastmilk supply (12). Further, newborns whose mothers have died through childbirth or shortly after delivery lack access to mother's breastmilk. In Ghana, an estimated 776 mothers passed away during childbirth or died as a result of childbirth related complications in 2020, and 838 maternal deaths were reported in 2019 (20). In light of these challenges, some women and families turn to milk sharing or wet-nursing to fulfill the need for breastmilk. A recent study examining milk sharing practices among Ghanaian women reports that 8% of the sample (*n* = 1050) indicated that they had ever shared milk with another lactating mother (21).

Recommendations and guidelines set forth by the World Health Organization (WHO) support the use of donor human milk (DHM) in the absence of mothers' own milk (MOM), especially for preterm infants and newborns with low birth weight (22). Thus, in situations where a mother's own milk is not available, such as in the event of low maternal supply, maternal death, illness, medication, disability or delayed lactation, human donor milk has been proved as the best substitute for infant feeding (23). Results from a clinical study after giving donor breastmilk to premature infants (under 37 weeks) at a hospital in Colombia revealed that those who did not receive the human milk had 4 times a greater chance of being intubated (OR 4.05; 95% CI 1.80–9.11). Again, it was found that receiving donor milk early- before the seventh day of life – reduced one's risk of developing necrotizing enterocolitis, intraventricular hemorrhage and sepsis (24).

Human milk banks (HMB) collect milk, process, screen, store, and distribute human milk to meet infants' specific needs. These banks are essential in the prevention of infant mortality and morbidity, especially, in low-income settings. Unfortunately, human milk banks have been established, primarily, in high-resourced settings, and areas most in need have limited access to such milk. Also, human milk banks have not been systematically scaled up (25), leading to important human milk availability and implementation gap(s). While human milk bank systems have been implemented in some low-and middle-income countries with great success in decreasing infant mortality and morbidity (26–28), efforts in Sub-Saharan Africa (SSA) have been limited despite the high rate of infant mortality and morbidity in countries in the sub-region. Currently, milk banks are available in two Sub-Saharan African countries, Kenya (29) and South Africa (30).

Ghana has implemented several maternal and child health programs to improve overall infant mortality rates, with specific attention toward promoting exclusive breastfeeding through the adoption of policies like Baby-Friendly Hospital Initiative (BFHI), National Breastfeeding Policy and Breastfeeding Promotion Regulation (31). It is imperative that healthcare professionals continue to advocate for exclusive breastfeeding and the use of human milk among mothers of infants with very low birth weight (VLBW). Research indicates that VLBW infants who receive breastmilk during the NICU hospitalization period have reduced incidence of preventable morbidities and re-hospitalization (32–34). It can also help children with developmental problems in infancy and childhood to overcome issues associated with developmental delays (35). However, there is still the need for DHM to support preterm babies and babies who may have lost their mothers during or after childbirth as well as mothers who are unable to generate their own breast milk after the birth of their babies.

In view of the above discussion, there is academic and political interest to incorporate human milk banks into the existing breastfeeding efforts. Hence, the purpose of the current study was to assess Ghanaian women's perspectives on HMB and their willingness to donate if one became available in the country.

## 2. Methods

### 2.1. Study design and participants

This mixed methods study recruited Ghanaian women aged 18 years and older to find out about the studied participants' views on Human Milk banking needs in Ghana. Based on the purpose of the current study, recruitment and data collection focused on the primary stakeholders, Ghanaian women both current and prospective mothers, who are involved in infant feeding. Stakeholders involved in infant feeding refers to currently pregnant women, lactating women, mothers with past breastfeeding experience, prospective mothers and women whose occupation involved the provision of any type of infant feeding support and counseling of Ghanaian women. The study was approved by the Indiana University institutional review board in April 2022 and the Cape Coast Teaching Hospital Ethical Review Committee (CCTHERC) in Ghana in June 2022. The lead author developed the research questions after attending a workshop on establishing human milk banks, authoring papers on breastfeeding and conducting extensive review of literature review on human milk banking (see reference section 24–30).

Two data collection methods were used: (A) Paper and pencil method was used where trained research personnel helped with the distribution of the study questionnaire to participants to complete. (B) An online survey method was used and distributed via an anonymous link to the study Qualtrics survey using social media platforms such as an “all-female Ghanaian support group”, ‘Tell It Moms' and other Facebook groups. We recruited additional participants through the snowball sampling method, where respondents who had completed the survey were asked to help recruit additional participants within their social networks. All surveys were in English, the official language of Ghana. We used a two-pronged strategy to ensure maximum reach of respondents. First, owing to the relative flexibility that online platforms offer, we were able to recruit from all 14 regions of the country using online web-driven mechanism. Additionally, recognizing the limitations of web-only recruitment efforts that excludes respondents without access to internet, we used paper and pencil approaches to facilitate the inclusion of additional voices. Thus, our sample was diverse in composition and was representative of the Ghanaian population. The questionnaire was pilot tested with twenty respondents and minor changes that did not affect the content were made to the questionnaire.

Respondents of the study were enlightened about the study and received clear explanation of the purpose of the study. No incentive was given for participating in the study. Closed and open-ended questions that inquire about the possibility of establishing a Human Milk Bank, willingness to donate breastmilk to the bank if one was established, the benefit of the human milk to children as well as the benefit of donating human milk were used in the study.

### 2.2. Data collection

Three of the authors and two research assistants administered the questionnaire. We received a total of 1,270 (*n* = 1270) responses. The questionnaires were collated and coded. Data screening was done to identify missing values (incomplete answers to all the questions) as well as outliers; missing data and outliers were excluded from data analysis (*n* = 321). We retained a final sample of nine hundred and forty-nine (*n* = 949) complete responses to the variables of interest and subsequent open-ended follow-up items.

For the quantitative analysis, six variables were included in this study. Specifically, we assessed participants' educational level (education), respondent willingness to donate to the Human Milk Bank (donate), feasibility of donating excess milk to the bank (feasible), perceptions that human milk banking will improve infant feeding (feeding), opinions on whether human milk banking will favor respondent's child (favor child) and respondents' perceptions on whether Ghana is ready for a human milk bank (ready).

### 2.3. Assessment measures

#### 2.3.1. Dependent variables (ready and donate)

The current study examined associations between 4 predictor variables on 2 out-comes: *Ready and Donate*. Both outcome variables were treated as binary variables. Thus, we asked the questions:

i. In Africa, human milk banks are located in Kenya and South Africa. In your view, do you think Ghana is ready to have Human Milk Bank?
ii. Do you think you will be willing to donate breastmilk to the Milk Bank in case there is one in your community?

Response options were “yes” coded as 1 and “No” coded as 0. Respondents were presented with a brief definition of human milk banks prior to being presented with questions. Further, participants were asked to provide explanations for their response choices. A text box option was provided to accommodate respondent explanations.

#### 2.3.2. Independent variables

We assessed associations for participant education, feasibility of milk donation, if human milk banks will improve infant feeding and whether human milk banking will favor one's child. These factors have been shown to be associated with perceptions about human milk banking in previous studies (36, 37).

#### 2.3.3. Statistical analysis (quantitative data analysis)

We computed frequencies and proportions for each of the study variables. We additionally conducted Pearson chi-square tests to examine if there were significant differences in responses for participants who responded YES or NO to the question (a) Is Ghana ready for a Human Milk Bank? And for participants who responded YES or NO to the question (b) Do you think you will be willing to donate breastmilk to the Milk Bank? Pearson chi-square tests were conducted between each independent study variable i.e. education, feasible, feeding, favor child and the two study outcome variables i.e., ready and donate. Logistic regression analysis was used to examine the independent associations between the outcome variables (ready and& donate) and all the independent variables in the model. The threshold for declaring statistical significance is a *p*-value < 0.05. All analyses were performed in R version 4.2.1 (R Core team, 2022; Boston, MA, USA).

### 2.4. Data analysis of the text (qualitative analysis)

Using an inductive and semantic approach, the researchers allowed the data to determine the themes by analyzing plainly the content of the data. The thematic analysis was performed following Braun and Clarke's (38) six steps of reading and re-reading the text to become familiar with the data, generating initial codes, searching for themes, re-viewing of themes, defining the themes and finally writing-up the report (38). Coding was done by two of the authors. Determination of inter-coder reliability was done using Holsti (39) coefficient (39). All the cases were coded by the two coders and because the quotes were short and straight forward, there was no disagreement between the coders. We therefore achieved an inter-coder reliability of one (1) which is a complete agreement between the coders indicating a very good reliability (39). To establish trustworthiness of the study, an external auditor who is an expert in qualitative research confirmed the research process as well as the results in order to ensure credibility and dependability. Detailed description of the research process has also been provided to ensure transferability. It is important to note that even though two or three excerpts may be cited to exemplify or elucidate a theme or sub-theme, such excerpts are chosen from many similar excerpts or occurrences in the data and thus have functional validity for the entire data.

## 3. Results

### 3.1. Quantitative results

Characteristics of the study sample are presented in Table 1. Overall, 64.7% of the study sample responded that Ghana is ready for a human milk bank. More than 70% of respondents were willing to donate their milk and about 69% believed that donating to the milk bank would favor their child. Additional characteristics are shown in Table 1.

**Table 1:** Characteristics of the study sample showing proportions and percentages for participants' education level, feasibility and benefits of milk donation, willingness to donate milk and Ghana's readiness for a HMB (*n* = 949).

	**Overall n (%)**
**Is Ghana ready for a HMB?**	
Yes	614 (64.7)
No	335 (35.3)
**Willing to donate milk to HMB**	
Yes	733 (77.2)
No	216 (22.8)
**Education**	
High school or less	219 (23.1)
College-level	541 (57.0)
Graduate	189 (19.9)
**Feasible to donate to HMB**	
Yes	818 (86.2)
No	131 (13.8)
**Donating to HMB will favor my child**	
Yes	659 (69.4)
No	290 (30.6)
**HMB will address infant feeding**	
Yes	779 (82.1)
No	170 (17.9)

All values are expressed as *n* (%). (%), Percent; HMB, Human Milk Bank.

At the bivariate level, respondent education (*p* = 0.002), feasibility to donate milk (*p* < 0.001), perception that donating to HMB will favor their child (p < 0.001), and the belief that HMB will address infant feeding (*p* < 0.001) were significantly associated with the outcome variables ready and donate (Table 2).

**Table 2:** Bivariate results showing associations between participants' education level, feasibility and benefits of milk donation with willingness to donate milk and Ghana's readiness for a HMB (*n* = 949).

	**Is Ghana ready for a HMB**			**Willing to donate milk to HMB**		
	**Yes n (%)**	**No n (%)**	**p**	**Yes n (%)**	**No n (%)**	**p**
Education			0.002^*^			<0.001^**^
High school or less	120 (19.5)	99 (29.6)		108 (14.7)	111 (51.4)	
College-level	320 (59.0)	179 (53.4)		458 (62.5)	83 (38.4)	
Graduate	132 (21.5)	57 (17.0)		167 (22.8)	22 (1023)	
Feasible to donate to HMB			<0.001^**^			<0.001^**^
Yes	576 (93.8)	242 (72.2)		694 (94.7)	124 (57.4)	
No	38 (6.2)	93 (27.8)		39 (5.3)	92 (42.6)	
Donating to HMB will favor my child			<0.001^**^			<0.001^**^
Yes	484 (78.8)	175 (52.2)		588 (80.2)	71 (32.9)	
No	130 (21.2)	160 (47.8)		145 (19.8)	153 (67.1)	
HMB will address infant feeding			<0.001^**^			<0.001^**^
Yes	573 (93.3)	206 (61.5)		655 (89.4)	124 (57.4)	
No	41 (6.7)	129 (38.5)		78 (10.6)	92 (42.6)	

All values are expressed as *n* (%). (%), Percent; HMB, Human Milk Bank. *P*-values are derived from Pearson's Chi-square test ^*^p < 0.05; ^**^p < 0.01.

#### 3.1.1. Is Ghana ready for HMB

Logistic regression results revealed no statistical differences between the 3 groups of respondent educational level regarding opinions about whether Ghana is ready for a human milk bank (Table 3). Respondents who reported that it is feasible to donate to HMB (OR, 2.36, CI 1.45, 3.88; *p* < 0.001), donating to HMB would favor child (OR, 1.92, CI 1.37, 2.68; *p* < 0.001) and HMB would address infant feeding (OR, 5.71, CI 3.81, 8.69; *p* < 0.001) all had higher odds of thinking that Ghana is ready for HMB (Table 3).

**Table 3:** Predictors of Ghana's readiness for and willingness to donate toward a HMB (*n* = 949).

	**Is Ghana ready for a HMB**		**Willing to donate milk to HMB**	
	**OR (95% CI)**	**P**	**OR (95% CI)**	**P**
**Education**				
High school or less	Reference		Reference	
College-level	0.99 (0.67, 1.45)	0.956	4.97 (3.23, 7.72)	<0.001^**^
Graduate	1.14 (0.71, 1.84)	0.577	7.20 (3.99, 13.49)	<0.001^**^
**Feasible to donate to HMB**				
Yes	2.36 (1.45, 3.88)	<0.001^**^	3.46 (2.10, 5.73)	<0.001^**^
No	Reference		Reference	
**Donating to HMB will favor my child**				
Yes	1.92 (1.37, 2.68)	<0.001^**^	5.76 (3.84, 8.72)	<0.001^**^
No	Reference		Reference	
**HMB will address infant feeding**				
Yes	5.71 (3.81, 8.69)	<0.001^**^	2.33 (1.46, 3.69)	<0.001^**^
No	Reference		Reference	

All values are expressed as *n* (%). (%), Percent; HMB, Human Milk Bank; OR, Odds Ratio; CI, Confidence Interval. ^*^p < 0.05; ^**^p < 0.01.

#### 3.1.2. Willing to donate milk to HMB

On willingness to donate milk to a HMB if such a bank was established, respondents who have college level education (OR, 4.97, CI 3.23, 7.72; *p* < 0.001) and graduate education (OR, 7.20, CI 3.99, 13.49; *p* < 0.001) were more likely to be willing to donate to HMB. Women who believe it is feasible to donate to HMB (OR, 3.46, CI 2.10, 5.73; *p* < 0.001), donating to HMB will favor child (OR, 5.76, CI 3.84, 8.72; *p* < 0.001) and HMB will address infant feeding (OR, 2.33, CI 1.46, 3.69; *p* < 0.001) all had higher odds of willingness to donate milk to the bank compared with respondents who indicated “no” in each variable (Table 3).

### 3.2. Qualitative results

In our sample, Six hundred and twenty-two (622) respondents provided explanations of their willingness to donate breastmilk to the milk bank. Ninety-four (94) respondents provided their explanations for their unwillingness to donate breastmilk to the milk bank. Four hundred and fifty-six (456) respondents believed that donating breast milk to a Human milk Bank would favor their children whereas one hundred and fifty-eight (158) respondents believed that donating breast milk to a Human milk Bank would not favor their children.

**Item:** *You indicated you would be willing to donate breastmilk to the milk bank. Please share why you chose this option*. Six hundred and twenty two (622) respondents provided their explanations.

#### 3.2.1. Reasons for willingness to donate to HMB

Respondents who indicated their willingness to donate breast milk to the Milk Bank in case there is one in their community to help preterm babies and babies whose mothers may have passed away were asked to provide their reasons. Four themes emerged as the reasons for indicating their willingness to donate: Breast milk being the best food for babies, Opportunity to help, Milk bank Project being good, and Formula being expensive.

*i. Breast milk being the best food for babies*

Most of the respondents acknowledged the important role breast milk plays in the growth of babies. Two excerpts are cited below for exemplification:

“*Breast milk is the best food for all babies but unfortunately not all babies get to have it. Some lose their mothers; some mothers are not able to breastfeed so they end up giving formula feeds. A breast milk bank will help solve this problem”*.

“*Artificial formula at early stages of a child's life is not the best. To me, breast milk is God's own natural way and it is the best of the best”*.

*ii. Opportunity to help*

Respondents also ascribed their willingness to donate to a milk bank to the opportunity they will get to be of help to humanity. Three such excerpts are cited below:

“*[I will donate] because I get to help someone, which feels good”*

“*[I will donate], if I am capable of helping another human to survive, why not”*

“*If at that particular time I am lactating, donating breast milk for a baby to be fed will be a joy and will be fulfilling to know a child is fed because I could provide it”*.

*iii. Project being good*

Some respondents also indicated that they were willing to donate because they believed a human milk bank is a good project. Some shared these:

“*At least it's a good project that will help save life”*.

“*[I will donate] because when I gave birth, at the initial state my breast milk was not available. So, I would have been happy if that option [of a human milk bank] existed or was available in Ghana”*.

*iv. Formula being expensive*

Some respondents also felt that donating to a milk bank will be helpful because the baby formula is expensive. Two studied participants shared these:

“*[I will donate] because my baby was at the neonatal intensive care unit and I saw how preterm babies and some twins that lost their mother after birth suffered. The formula was too expensive for their family to afford”*.

“*[I will donate] because there are babies who are suffering from acute malnutrition just because they lost their mothers at birth and family are unable to afford baby food or not able to mix the food in the right texture”*.

**Item:** *You indicated you would not be willing to donate breastmilk to the milk bank. Please share why you chose this option*. Ninety-four respondents provided their explanations.

#### 3.2.2. Reasons for not willing to donate to HMB

Even though a majority of the respondents were willing to donate to a milk bank, those respondents who indicated that they were not willing to donate to a human milk bank also provided their reasons and four themes emerged. The themes are: Human Milk Bank concept being strange and/or bizarre, Fear of infections, Religious beliefs and Insufficient information.

*i. Human Milk Bank concept being strange and/or bizarre*

The major reasons provided revolved around participant discomfort with human milk banks. Participants perceived HMB as strange and bizarre. Three respondents' responses are cited below:

“*It just seems weird, I have never thought of that. It would take effort for me to do that”*.

“*For now, it sounds weird”*.

“*It is just a bit awkward and new. It will need sometime for people to get used to the idea”*.

*ii. Fear of infections*

Some of the respondents expressed fear and concerns about the risk of infection from a donated human milk and its consequences. Three respondents noted:

“*[I will not donate] due to risk of infections. Moreover, there are other alternatives”*.

“*I believe that whatever that is in it [the breast milk] will be transferred to the child”*.

“*I am worried about hygienic conditions and knowing how what one eats affects the quality of breast milk production”*.

*iii. Religious beliefs*

Some respondents linked their religious beliefs as the reason why they were neither able nor willing to donate to a milk bank. There excerpts are cited below for exemplification:

“*Per my religion any child you breastfeed with same breast milk they automatically become brothers or sisters. They are not allowed to marry and how will I know who takes my breast milk”*.

“*It is for spiritual reasons”*.

“*It's against my beliefs”*.

*iv. Not much information*

Some respondents also indicated that they were not willing to donate human milk to the human milk bank because they did not know much about the idea of a human milk bank. The following are some of their narratives:

“*I do not have much information on this”*.

“*Am afraid and I do not know the implications”*.

“*I would want to read more on it”*.

**Item:** *You indicated that you believe donating breast milk to a Human milk Bank will favor your child. Please explain why you chose this option*. Four hundred and fifty-six (456) respondents provided their explanations.

#### 3.2.3. Reasons for believing that donating to HMB will favor your child

Respondents were asked to explain why they indicated that they believed donating to a Human milk bank will favor their child. Out of the various reasons provided, two main themes emerged. The themes were: Abundance of available breastmilk to feed infants and Readily available breast milk.

*i. Abundance of available breastmilk*

Most of the respondents ascribed their reason for believing that donating to the milk bank will favor their child to the fact that there will be more production of milk for their child to feed on. Two had this to say:

“*I would have to express more milk in order to be able to donate. The more I express, the more I get, so my baby will have more to feed”*.

“*Breastfeeding is a demand and supply kind of. The more I express some down, the more I produce milk for my baby”*.

*ii. Readily available breast milk*

Some of the respondents indicated that due to an unforeseen event that could prevent them from continuing to breast-feed their children, by donating milk to the milk bank, there would be readily available breast milk for their child to benefit from. Two studied participants shared these:

“*Because at any point in time if due to health reasons am not able to breastfeed, there will always be a readily available alternative which is the human milk bank”*.

“*Because in future when I do not have enough [breast milk], I can always reach out to the milk bank for support”*.

**Item:** *You indicated that you DO NOT believe that donating breast milk to a Human milk Bank will favor your child. Please explain why you chose this option*. One hundred and fifty eight (158) respondents provided some explanations.

#### 3.2.4. Reasons for believing that donating to HMB will NOT favor your child

Respondents were asked to explain why they indicated that they believed donating to a Human milk bank will not favor their child. Two themes emerged: No direct effect on them for donating to the HMB; and Donation to the HMB leading to reduced amounts of breastmilk for their child(ren).

### 3.3. No direct effect

Respondents in this category felt that donating to a milk bank will not have any direct favor on their child. Two study participants shared the excerpts below:

“*Because donating my breast milk has no direct effect on him [my child].”*

“*My child doesn't stand to benefit from the milk I've donated in anyways unless it generates income for me”*.

### 3.4. Reduced supply

Some of the respondents explained that they did not believe they would have enough breastmilk for their own child when they donate to the milk bank. Two excerpts shared by two of the studied participants are cited below:

“*If it is after 6 months when I am done with exclusive breastfeeding I could try, other than that I will not want to reduce the supply for my baby”*.

“*I do not produce too much milk at a time so donating whiles breastfeeding may leave just a little milk for my child”*.

## 4. Discussion

The findings of the current study indicate that majority of the respondents are in favor of having a milk bank in their country. These findings support the use of donor human milk as the best substitute for mother's own milk when the latter is unavailable. We learn further, that, respondents saw the HMB proposition as an opportunity to engage in a humanitarian act such as helping preterm babies and babies who may have lost their mothers after birth and hence an opportunity to assist less fortunate infants. In addition, the respondents viewed HMBs as lifesaving for infants cognizant of the fact that access to donor milk will help mitigate or eliminate morbidity and mortality associated to lack of efficacious infant nutrition. This finding is consistent with those of several other studies that document the benefits of donor human milk on newborns' development (23, 40). For example, breastfeeding and the use of human milk has been shown to have the best impact on child survival (41). Furthermore, in Brazil, breastfeeding promotion and support, including the use of donor human milk from human milk banks, has contributed substantially to reduce its infant mortality (26).

From our findings, respondents believed that donating to HMB will create abundance of breastmilk for their own children given the fact that increased breastmilk production was a result of constant and consistent breastmilk expression (42). The implication of this finding is that it sends a positive signal about the likelihood that breastfeeding mothers who are able to produce breastmilk in high volumes will be willing to donate freely to HMB when they are established in Ghana. Respondents also counted on breast milk becoming readily available at the HMB if they and others donate to the bank. Thus, they saw donating to HMB as a win-win situation, first for themselves in terms of increased breastmilk production, and donor breastmilk being available at the NICU in the event that they delivered a preterm or low birthweight infant.

The findings suggest that attaining a higher educational level (college and graduate education) resulted in a possible acceptance and willingness to donate breastmilk to HMB which is supported by previous related studies (43, 44). Nevertheless, we observed that some respondents reported having limited information about HMB. Again, responses provided by women who expressed their unwillingness to donate breastmilk due to the perception of a reduction in supply for their own baby is an indication that some women lack understanding of how milk production works. This creates an opportunity to scale up lactation and breastfeeding education for women of all educational levels. Lactation education focusing on the benefits of increasing milk production is important, not only for the purpose of donating to the HMB, but for the babies of the donating mothers themselves. To this effect, existing breastfeeding campaigns must invest in educational programs that target lactating mothers at postnatal care or child welfare centers. Information presented at these educational programs should be evidence-based, stemming from rigorous studies that have established the benefits of breast milk production for baby and milk donation for other vulnerable infants.

The reasons given for unwillingness to donate to HMB such as the concept being strange and/or bizarre and religious beliefs is supported by previous studies (45). This finding may be indicative of the need to explore further the socio-cultural factors and religious concerns that could act as barriers to the acceptability of HMB. The fear of possible risk of infections being transmitted to babies, a reason given by some respondents not willingly to donate breastmilk to HMB corroborates with reports from similar studies (36, 46–48). These findings imply that more sensitization and health education programs may be required to allay the fears of the general population. Campaigns must use culturally acceptable forms of communication and appropriate communication channels such as popular media and key community leaders to gain member buy-in (49). Additionally, using gender-inclusive language which promotes human milk banking as integral to exclusive breastfeeding and a strategy that facilitates the wellbeing of the entire family, not just the prerogative of women and children is key (49). Again, our findings suggest that some Ghanaian mothers would be willingly to donate their milk when there is an assurance of addressing logistics challenges in health facilities in the event of establishing HMBs in Ghana. Addressing logistic constraints include measures to ensure the safe handling and screening of donated breastmilk, effective storage/preservation processes to guarantee that the nutritional quality and microbiological safety requirements are met in HMBs.

### 4.1. Limitations

This study is not without some limitations. Our study participants were recruited through the snowball method where recruited participants helped to recruit others for the study. This recruitment strategy meant participants may have likely recruited co-workers, family members, and friends who might have similar experiences and beliefs. However, the benefit of gaining access to the population and gaining their trust was worth the use of this research methodology. Another limitation stems from the study questionnaire being provided in English only, and this may have deterred potential participants who could not read and write in English from participating. Additionally, participants who used the paper and pencil method of completing the survey received translation and interpretation. This approach may have subjected participant responses to social desirability bias compared to the participants who used the online Qualtrics survey where respondents received no translation. Further, our survey did not provide participants a detailed description of what donating breastmilk to the HMB would entail. This may have influenced participant responses due to limited information to make an informed decision about HMB. Another limitation of the study is the high number of missing data and outliers. There was a large number of respondents who answered parts of the survey but did not complete the entire questionnaire. The missing responses could have impacted the outcome of the study. Furthermore, a potential limitation of the current study stems from the inclusion of women who had no prior experience with breastfeeding. This strategy however ensured that we gained prevailing perceptions of HMB among Ghanaian women from representative of the entire female population. Despite these limitations, this research is important as it sheds light on acceptance of a Human Milk Banking system in Ghana to help improve childhood nutrition.

### 4.2. Implications of the current study

While majority of our respondents support human milk bank and are willing to donate to the breast milk bank if one exists in their community, some respondents expressed concerns about fear of possible infection due to improper hygiene. Coutsoudis et al. (50) reports that in regions where HIV is prevalent, infant feeding choices are often stigmatized and feared due to the association with the disease (50). There is no doubt that proper screening of donated milk will minimize the risk of infection associated with donated milk. Other respondents additionally commented on the religious implications of feeding donated milk to their children. This finding is also reported by other scholars in which they noted that religious considerations prohibit siblings (children receiving breastmilk from same mother) from “intermarriage” in the Islam religion; this also presents a challenge to donating and receiving donor human milk (51, 52). The implications of the findings underscores the need to keep a record of milk donors and recipients to effectively address the religious concerns (52). Another suggestions is to incorporate a system of single donor networks, where the donor and recipients know each other's identities (52). Furthermore, findings from our research are supported by findings from other studies that show that some African women and health professionals lack information on HMB and have little or no knowledge about it (36, 53–55). The lack of knowledge suggests that it is important to engage in a mass education of the Ghanaian public when establishing the human milk bank; this is because with proper education will come acceptance and support from mothers and the public.

From this study, we identified gaps that need to be filled in order to take definitive steps toward establishing a human milk bank in Ghana. First, there is no nationally available data on the proportion of preterm deliveries occurring annually (13). Again, there is little information about how preterm and low birthweight babies in the NICU are fed. Further, in situations of delayed maternal lactation or maternal death, data is lacking on breastfeeding practices for orphaned babies. While there is evidence of peer to peer (informal) milk donation and wet nursing among Ghanaian women (21), there is no published data on whether sick, preterm and LBW neonates are fed donated human milk in the NICU. Obtaining this information will be critical to fully assess the need for establishing HMB in the country.

In this regard, we suggest that futures studies investigate the current feeding practices in Ghanaian NICUs. The Ghana Statistical Service, together with the ministry of Health, must invest in developing a comprehensive surveillance system where national estimates of preterm deliveries and (very) low birthweight neonates are recorded and monitored. Again, future studies may explore the practice of wet-nursing/ informal milk sharing in the Ghanaian community to augment breastfeeding rates and human milk feeding for newborns.

Other studies may investigate women's preferences between HMB and wet-nursing/direct milk sharing, with specific focus on uncovering myths and misconceptions to be used during targeted breastfeeding and human milk educational campaigns.

## 5. Conclusion

In conclusion, this study has shown support for the building of HMB; an initiative that research has shown to be good for preterm babies and babies who may have lost their mothers during or after childbirth (as noted in the qualitative responses) as well as mothers who are unable to generate their own breast milk after the birth of their babies. HMB can be used as a short-term passage for mothers to build up their own milk that is appropriate for their own child. Thus, the studied Ghanaian citizens' perspectives to have a Human Milk Bank will enable stakeholders to make Human Milk banking an essential part of the ongoing breastfeeding promotion in Ghana. This will help to reduce infant mortality rate in preterm and low-birthweight infants.

## Data availability statement

The raw data supporting the conclusions of this article will be made available by the authors, without undue reservation.

## Ethics statement

The studies involving human participants were reviewed and approved by the Indiana University Institutional Review Board and the Cape Coast Teaching Hospital Ethical Review Committee. The patients/participants provided their written informed consent to participate in this study.

## Author contributions

CO: conceptualized the study. CO, FJ, SA-E, CN-A, CP, IG, and EO-G: drafted the initial version of the manuscript, data analysis, and data curation was done by CO. SA-E, CN-A, and FJ: editing and review was performed by CO, FJ, and EO-G. CO, FJ, SA-E, CN-A, CP, IG, and EO-G reviewed the final draft of the manuscript. All authors read and approved the final manuscript.

## References

[1] Macrotrends. World Infant Mortality Rate 1950-2023. (2023). Available online at: https://www.macrotrends.net/countries/WLD/world/infant-mortality-rate (accessed March 30, 2023).

[2] UNICEF. Neonatal mortality. UNICEF DATA. (2023). Available online at: https://data.unicef.org/topic/child-survival/neonatal-mortality (accessed March 30, 2023).

[3] World Health Organization. Newborn Mortality. (2022). Available online at: https://www.who.int/news-room/fact-sheets/detail/levels-and-trends-in-child-mortality-report-2021 (accessed July 18, 2023).

[4] Macrotrends. Ghana Infant Mortality Rate 1950-2023. 2023. (2022). Available online at: https://www.macrotrends.net/countries/GHA/ghana/infant-mortality-rate (accessed March 30, 2023).

[5] UNICEF. Ghana (GHA) - Demographics, Health & Infant Mortality. UNICEF DATA. (2022). Available online at: https://data.unicef.org/country/gha (accessed September 6, 2023).

[6] Ghana Statistical Service,. Demographic Health Survey 2014. Rockville, Maryland, USA: Ghana Health Service (GHS) ICF International. (2015). Available online at: https://dhsprogram.com/pubs/pdf/fr307/fr307.pdf (accessed August 6, 2023).

[7] Adjei G Darteh EKM Nettey OEA Doku DT. Neonatal mortality in the central districts of Ghana: analysis of community and composition factors. BMC Public Health. (2021) 21:173. 10.1186/s12889-021-10156-6 33478435 PMC7819257

[8] Healthy Newborn Network,. Ghana launches a national newborn health strategy action plan to speed up the reduction of newborn deaths. Healthy Newborn Network. (2014). Available online at: https://www.healthynewbornnetwork.org/blog/ghana-launches-national-newborn-health-strategy-action-plan-speed-reduction-new-born-deaths/ (accessed November 4, 2022).

[9] Diedrich CM,. Neonatal Mortality in Ghana. (2016). Available online at: https://scholarworks.gvsu.edu/cgi/viewcontent.cgi?article=1493&context=honorsprojects (accessed November 4, 2022).

[10] UNICEF. Newborn Health. (2016). Available online at: https://www.unicef.org/ghana/newborn-health (accessed November 4, 2022).

[11] Dare S Oduro AR Owusu-Agyei S Mackay DF Gruer L Manyeh AK. Neonatal mortality rates, characteristics, and risk factors for neonatal deaths in Ghana: analyses of data from two health and demographic surveillance systems. Glob. Health Action. (2021) 14:1938871. 10.1080/16549716.2021.1938871 34308793 PMC8317945

[12] Bliss K,. Improving Newborn health in Ghana. Strengthening Services in the Center Periphery. CSIS Global Health Policy Center. (2016). Available online at: https://vision2017.csis.org/strengthening-services-center-periphery-improve-newborn-health-ghana/../strengthening-services-center-periphery-improve-newborn-health-ghana/index.html (accessed November 4, 2022).

[13] Axame WK Binka FN Kweku M. Prevalence and factors associated with low birth weight and preterm delivery in the ho municipality of Ghana. Adv public health. (2022) 2022:e3955869. 10.1155/2022/3955869

[14] Abubakari A Kynast-Wolf G Jahn A. Maternal determinants of birth weight in northern Ghana. PLoS ONE. (2015) 10:e0135641. 10.1371/journal.pone.0135641 26281013 PMC4539219

[15] Fosu MO Abdul-Rahaman I Yekeen R. Maternal risk factors for low birth weight in a District Hospital in Ashanti Region of Ghana. Res Obstet Gynaecol. (2013) 2:48–54.

[16] Adu-Bonsaffoh K Gyamfi-Bannerman C Oppong SA Seffah JD. Determinants and outcomes of preterm births at a tertiary hospital in Ghana. Placenta. (2019) 79:62–7. 10.1016/j.placenta.2019.01.007 30654915

[17] Afeke I Mac-Ankrah L Jamfaru I Amegan-Aho KH Mbroh HK Lokpo SY. Maternal age, low birth weight and early neonatal death in tertiary hospital in the volta region of Ghana. Open J Pediatr. (2017) 7:254. 10.4236/ojped.2017.74029

[18] O'Leary M Edmond K Floyd S Newton S Thomas G Thomas SL. cohort study of low birth weight and health outcomes in the first year of life, Ghana. Bull World Health Organ. (2017) 95:574–83. 10.2471/BLT.16.180273 28804169 PMC5537746

[19] Vesel L Manu A Lohela TJ Gabrysch S Okyere E Asbroek AHA. Quality of newborn care: a health facility assessment in rural Ghana using survey, vignette and surveillance data. BMJ Open. (2013) 3:e002326. 10.1136/bmjopen-2012-002326 23667161 PMC3651975

[20] World Health Organization Ghana. Ghana holds Conference on Maternal, Child Health and Nutrition. WHO | Regional Office for Africa. (2021). Available online at: https://www.afro.who.int/news/ghana-holds-conference-maternal-child-health-and-nutrition (accessed March 30, 2023).

[21] Obeng C Jackson F Nsiah-Asamoah C Amissah-Essel S Obeng-Gyasi B Perry CA. Human milk for vulnerable infants: breastfeeding and milk sharing practice among Ghanaian women. Int J Environ Res Public Health. (2022) 19:16560. 10.3390/ijerph192416560 36554441 PMC9779609

[22] World Health Organization UNICEF. Global strategy for infant and young child feeding. Geneva: WHO. (2003). Available online at: https://apps.who.int/iris/bitstream/handle/10665/42590/9241562218.pdf;jsessionid=6F7537A2B82ED3775BE5F7A4DD406C8A?sequence=1 (accessed November 7, 2023).

[23] Haiden N Ziegler EE. Human Milk Banking. Ann Nutr Metab. (2016) 69:7–15. 10.1159/000452821 28103607

[24] Torres-Muñoz J Jimenez-Fernandez CA Murillo-Alvarado J Torres-Figueroa S Castro JP. Clinical results of the implementation of a breast milk bank in premature infants (under 37 weeks) at the hospital universitario del valle 2018–2020. Nutrients. (2021) 13:2187. 10.3390/nu13072187 34202034 PMC8308280

[25] Demarchis A Israel-Ballard K Mansen K Engmann C. Establishing an integrated human milk banking approach to strengthen newborn care. J Perinatol. (2016) 10:37. 10.1038/jp.2016.198 27831549 PMC5415705

[26] Pimenteira Thomaz AC Maia Loureiro LV da Silva Oliveira T de Mendonça Furtado Montenegro NC Dantas Almeida Júnior E Fernando Rodrigues Soriano C. The human milk donation experience: motives, influencing factors, and regular donation. J Hum Lact. (2008) 24:69–76. 10.1177/0890334407310580 18281358

[27] Bhasin M Nangia S Goel S. Role of human milk banks amid COVID 19: perspective from a milk bank in India. Int Breastfeed J. (2020) 15:104. 10.1186/s13006-020-00346-0 33267891 PMC7709091

[28] Human Milk Bank Network Southeast Asia. Minimum Standards for the Establishment and Operation of Human Milk Banks in Southeast Asia. (2021). Available online at: http://www.aliveandthrive.org/en/resources/minimum-standards-for-the-establishment-and-operation-of-human-milk-banks-in-southeast-asia (accessed July 19, 2022).

[29] County Innovation Challenge Fund. Helping Babies thrive: Establishing the first Integrated Human Milk Banking System in Kenya. (2019). Available online at: https://options.co.uk/sites/default/files/helping_babies_thrive_hmb.pdf (accessed July 19, 2022)

[30] Biggs C. Talking the Talk but not walking the walk: donating to human milk banks in South Africa. J Hum Lact. (2021) 37:105–13. 10.1177/0890334420970495 33201758

[31] Aryeetey R Harding K Hromi-Fiedler A Pérez-Escamilla R. Analysis of stakeholder networks for breastfeeding policies and programs in Ghana. Int Breastfeed J. (2020) 15:74. 10.1186/s13006-020-00311-x 32831116 PMC7444079

[32] Corpeleijn WE Kouwenhoven SMP Paap MC Vliet I. van, Scheerder I, Muizer Y, et al. Intake of own mother's milk during the first days of life is associated with decreased morbidity and mortality in very low birth weight infants during the first 60 days of life. Neonatology. (2012) 102:276–81. 10.1159/000341335 22922675

[33] Vohr BR Poindexter BB Dusick AM McKinley LT Higgins RD Langer JC. Persistent beneficial effects of breast milk ingested in the neonatal intensive care unit on outcomes of extremely low birth weight infants at 30 months of age. Pediatrics. (2007) 120:e953–9. 10.1542/peds.2006-3227 17908750

[34] Patel AL Johnson TJ Engstrom JL Fogg LF Jegier BJ Bigger HR. Impact of early human milk on sepsis and health-care costs in very low birth weight infants. J Perinatol. (2013) 33:514–9. 10.1038/jp.2013.2 23370606 PMC3644388

[35] Spiegler J Preuß M Gebauer C Bendiks M Herting E Göpel W. Does breastmilk influence the development of bronchopulmonary dysplasia? J Pediatr. (2016) 169:76–80. 10.1016/j.jpeds.2015.10.080 26621048

[36] Kimani-Murage EW Wanjohi MN Kamande EW Macharia TN Mwaniki E Zerfu T. Perceptions on donated human milk and human milk banking in Nairobi, Kenya. Matern Child Nutr. (2019) 15:e12842. 10.1111/mcn.12842 31099159 PMC6859964

[37] African Population Health Research Center. Integrating Human Milk Banking with Breastfeeding Promotion and Newborn Care: is Kenya Ready? (2022). Available online at: https://aphrc.org/wp-content/uploads/2019/07/Human-Milk-Bank-Project-Briefing-Paper-APHRC.pdf (accessed August 22, 2022).

[38] Braun V Clarke V. Using thematic analysis in psychology. Qual Res Psychol. (2006) 3:77–101. 10.1191/1478088706qp063oa

[39] Holsti OR. Content analysis for the social sciences and humanities Reading, Mass. London: Addison-Wesley Pub Co. (1969).

[40] Simmer K. The knowns and unknowns of human milk banking early nutrition: impact on short- and long-term health nestlé. Nutr Inst Workshop Ser Pediatr program. (2011) 68:49–64. 10.1159/000325659 22044891

[41] Victora CG Bahl R Barros AJD França GVA Horton S Krasevec J. Breastfeeding in the 21st century: epidemiology, mechanisms, and lifelong effect. Lancet. (2016) 387:475–90. 10.1016/S0140-6736(15)01024-7 26869575

[42] Kent JC. How Breastfeeding Works. J Midwifery Womens Health. (2007) 52:564–70. 10.1016/j.jmwh.2007.04.007 17983993

[43] Esquerra-Zwiers A Rossman B Meier P Engstrom J Janes J Patel A. “It's somebody else's milk”: unraveling the tension in mothers of preterm infants who provide consent for pasteurized donor human milk. J Hum Lact. (2016) 32:95–102. 10.1177/0890334415617939 26590179 PMC4959541

[44] Jang HL Cho JY Kim M Kim EJ Park EY Park SA. The experience of human milk banking for 8 years: korean perspective. J Korean Med Sci. (2016) 31:1775–83. 10.3346/jkms.2016.31.11.1775 27709856 PMC5056210

[45] Karadag A Ozdemir R Ak M Ozer A Dogan DG Elkiran O. Human milk banking and milk kinship: Perspectives of mothers in a Muslim country. J Trop Pediatr. (2015) 61:188–96. 10.1093/tropej/fmv018 25828832

[46] Chagwena DT Mugariri F Sithole B Mataga SF Danda R Matsungo TM. Acceptability of donor breastmilk banking among health workers: a cross-sectional survey in Zimbabwean urban settings. Int Breastfeed J. (2020) 15:37. 10.1186/s13006-020-00283-y 32393361 PMC7216340

[47] Magowan S Burgoine K Ogara C Ditai J Gladstone M. Exploring the barriers and facilitators to the acceptability of donor human milk in eastern Uganda - a qualitative study. Int Breastfeed J. (2020) 15:28. 10.1186/s13006-020-00272-1 32303270 PMC7165402

[48] Mondkar J Chugh Sachdeva R Shanbhag S Khan A Manuhar Sinha M Dasgupta R. Understanding barriers and facilitators for human milk banking among service providers, mothers, and influencers of preterm and sick neonates admitted at two health facilities in a metropolitan city in India. Breastfeed Med. (2018) 13:694–701. 10.1089/bfm.2018.0103 30383389

[49] Dutta T Agley J Lin HC Xiao Y. Gender-responsive language in the National Policy Guidelines for Immunization in Kenya and changes in prevalence of tetanus vaccination among women, 2008–09 to 2014: A mixed methods study. Womens Stud Int Forum. (2021) 86:102476. 10.1016/j.wsif.2021.102476

[50] Coutsoudis I Petrites A Coutsoudis A. Acceptability of donated breast milk in a resource limited South African setting. Int Breastfeed J. (2011) 6:3. 10.1186/1746-4358-6-3 21342496 PMC3049132

[51] Ozdemir R Ak M Karatas M Ozer A Dogan DG Karadag A. Human milk banking and milk kinship: perspectives of religious officers in a Muslim country. J Perinatol. (2015) 35:137–41. 10.1038/jp.2014.177 25254333

[52] Khalil A Buffin R Sanlaville D Picaud JC. Milk kinship is not an obstacle to using donor human milk to feed preterm infants in Muslim countries. Acta Paediatr. (2016) 105:462–7. 10.1111/apa.13308 26659819

[53] Doshmangir L Naghshi M Khabiri R. Factors influencing donations to human milk bank: a systematic review of facilitators and barriers. Breastfeed Med. (2019) 21:14. 10.1089/bfm.2019.0002 30896254

[54] Abhulimhen-Iyoha BI Okonkwo IR Ideh RC Okolo AA. Mothers' perception of the use of banked human milk for feeding of the infants. Niger J Paediatr. (2015) 42:223–7. 10.4314/njp.v42i3.10

[55] Gelano TF Bacha YD Assefa N Motumma A Roba AA Ayele Y. Acceptability of donor breast milk banking, its use for feeding infants, and associated factors among mothers in eastern Ethiopia. Int. Breastfeed. J. (2018) 13:23. Available online at: https://doi-org.proxyiub.uits.iu.edu/10.1186/s13006-018-0163-z 29983728 10.1186/s13006-018-0163-z PMC6019314

